# Assessment of a dried blood spot C-reactive protein method to identify disease flares in rheumatoid arthritis patients

**DOI:** 10.1038/s41598-020-77826-0

**Published:** 2020-12-03

**Authors:** Leon G. D’Cruz, Kevin G. McEleney, Chris Cochrane, Kyle B. C. Tan, Priyank Shukla, Philip V. Gardiner, Dawn Small, Shu-Dong Zhang, David S. Gibson

**Affiliations:** 1 Northern Ireland Centre for Stratified Medicine (NICSM), Biomedical Sciences Research Institute, Ulster University, Altnagelvin Area Hospital, C-TRIC Building, Glenshane Road, Londonderry, BT47 6SB UK; 2 Fusion Antibodies Plc., Springbank Road, Springbank Industrial Estate, Belfast, UK; 3 Rheumatology Department, Altnagelvin Hospital, Western Health and Social Care Trust, Glenshane Road, Londonderry, BT47 6SB UK

**Keywords:** Biochemistry, Prognostic markers, Autoimmune diseases, Rheumatoid arthritis, Medical and clinical diagnostics

## Abstract

Rheumatoid arthritis (RA) is characterised by painful, stiff and swollen joints. RA features sporadic ‘flares’ or inflammatory episodes—mostly occurring outside clinics—where symptoms worsen and plasma C-reactive protein (CRP) becomes elevated. Poor control of inflammation results in higher rates of irreversible joint damage, increased disability, and poorer quality of life. Flares need to be accurately identified and managed. A method comparison study was designed to assess agreement between CRP values obtained by dried blood spot (DBS) versus conventional venepuncture sampling. The ability of a weekly DBS sampling and CRP test regime to detect flare outside the clinic was also assessed. Matched venepuncture and finger lancet DBS samples were collected from n = 100 RA patients with active disease at baseline and 6 weeks (NCT02809547). A subset of n = 30 RA patients submitted weekly DBS samples over the study period. Patient demographics, including self-reported flares were recorded. DBS sample CRP measurements were made by enzyme-linked immunosorbent assay, and venepuncture samples by a reference immunoturbometric assay. Data was compared between sample types by Bland–Altman and weighted Deming regression analyses. Flare detection sensitivity and specificity were compared between ‘minimal’ baseline and 6 week sample CRP data and the ‘continuous’ weekly CRP data. Baseline DBS ELISA assay CRP measures yielded a mean positive bias of 2.693 ± 8.640 (95% limits of agreement − 14.24 to 19.63%), when compared to reference assay data. Deming regression revealed good agreement between the DBS ELISA method and reference assay data, with baseline data slope of 0.978 and intercept -0.153. The specificity of ‘continuous’ area under the curve (AUC) CRP data (72.7%) to identify flares, was greater than ‘minimal’ AUC CRP data (54.5%). This study indicates reasonable agreement between DBS and the reference method, especially at low to mid-range CRP values. Importantly, longitudinal CRP measurement in RA patients helps to clearly identify flare and thus could assist in remote monitoring strategies and may facilitate timely therapeutic intervention.

***Trial registration***: The Remote Arthritis Disease Activity MonitoR (RADAR) study was registered on 22/06/2016 at ClinicalTrials.gov Identifier: NCT02809547. https://clinicaltrials.gov/ct2/show/NCT02809547.

## Introduction

Rheumatoid arthritis (RA) is a debilitating and chronic inflammatory disease. RA is a characterised by sporadic synovitis, or ‘flares’ during which disease activity increases and joints become more painful and swollen^1^. These flares can lead to progressive and irreversible damage to joints, loss of musculoskeletal function and increased disability^2^. RA is also characterised by widespread systemic inflammation leading to general physical and mental impairment, with concomitant worsening of quality of life^3^. Monitoring of flares in disease activity by surrogate biological markers of inflammation is therefore important in RA, to identify individuals susceptible to joint destruction. Continuous monitoring during periods of active disease could also facilitate more accurate decisions on dosage of drugs and when to switch from ineffective treatments.

Inflammation in the synovium, or joint lining, correlates with systemic inflammatory responses in RA^2,4^. Acute phase immune response markers such as C-reactive protein (CRP) and erythrocyte sedimentation rate (ESR) are typically measured to record inflammation at the biological level and track response to therapy. ESR and CRP correlate with radiographic progression and they have been incorporated into disease activity composite scores such as DAS28, which is used clinically as a tool to assess treatment efficacy and target remission^5,6^.

Collection of blood samples from RA patients on a regular basis for the monitoring of inflammation markers presents unique challenges. Since blood collection currently requires trained personnel, patients either need to travel to outpatient clinics with significant costs and inconvenience, or qualified phlebotomists need to schedule home visits. However, during disease flare a patient’s mobility can be severely limited and home based health service models may not be available or cost effective, particularly for rural populations.

The Remote Arthritis Disease Activity MonitoR (RADAR) study was therefore designed to test the reliability of a blood sampling method with potential to address these challenges. Patients with active RA were equipped to provide dried-blood spots (DBS) from home and return the samples to a clinical laboratory by standard postal service. The potential for DBS samples to assist routine diagnosis and clinical assessment is well established^7–9^. Some blood biomarkers, including CRP, are stable in DBS for up to a week’s storage at ambient temperatures and even longer periods at − 80 °C^10,11^.

This study investigates how accurate CRP concentration measurements from DBS are compared to traditional whole blood samples in 100 RA patients with active disease. The purpose was to provide data on the reliability of DBS as a potential platform to continuously and remotely monitor RA patients. Importantly, the ability of a weekly DBS monitoring regime to detect disease flares was investigated in 30 of the RA patients during a 6 week follow up period. As such this is the first study to provide evidence of DBS CRP utility in an active RA population.

## Methods

### Participant recruitment

The research team at Ulster University collaborated with rheumatologists from the Western Health and Social Care Trust (WHSCT) to design, conduct and recruit patients to the study. One hundred patients identified using the following inclusion/exclusion criteria were recruited into the prospective observational cohort study: Remote Arthritis Disease Activity MonitoR (RADAR); ClinicalTrials.gov Identifier: NCT02809547. Inclusion criteria: aged between 18–90 years, diagnosed with RA (according to American College of Rheumatology criteria^12^), diagnosed with RA for a minimum of 1 year and maximum 10 year duration, active disease flares on a regular basis, and receiving a conventional (non-biologic) disease modifying anti-rheumatic drug (DMARD). Patient reported flare was defined as elevated stiffness and/ or pain in one or more joints lasting more than 24 h. Exclusion criteria: any other inflammatory conditions, any infections or trauma during study period, and have restricted hand function (determined by clinical team). Office for Research Ethics Committees Northern Ireland (ORECNI) (16/NI/0039), Ulster University Research Ethics Committee (UREC) (REC/16/0019) and WHSCT (WT/14/27) approvals were obtained for the study. All methods were performed in accordance with the relevant guidelines and regulations. Informed consent was obtained from all participants enrolled to the study.

### Sample size calculations

A minimum sample size of 34 was required for 90% power, α = 0.05 and the ideal anticipated Cohen’s “d” effect size of 0.80, calculated using a two-sample t-test with SPSS ver. 25 (IBM Corp) integrated with R version 3.33. Thus, our sample size of 100 ensured adequate power to the study, in line with guidelines set by the Clinical and Laboratory Standards Institute EP09c-A3^13,14^. Additionally, the sample number ensured that normal variation in CRP concentrations observed within an active disease RA cohort were recorded to analyse the precision of the sampling methods. The range ratio of reference method CRP concentrations across the cohort at the 6 week time point was 421 (126.5 mg/L high: 0.3 mg/L low).

### Whole blood and DBS sample collection

Venepuncture whole blood samples collected as part of standard care were forwarded to the hospital laboratories for multiple tests including CRP. An additional 5 ml EDTA tube of blood and 5 finger lancet droplets of blood (approximately 10 μl each) were collected from all 100 RADAR study participants. Finger lancet blood droplets were deposited onto DBS Protein Saver 903™ cards (Whatman, GE Healthcare Life Sciences, Buckinghamshire, UK), pre-treated with a proprietary protein stabiliser coating. Both blood sample types were collected at study baseline and a 6 week follow up appointment at an outpatient rheumatology clinic for all participants. All patients received training in lancet and DBS card use at baseline and completed a Likert type questionnaire^15^, at the 6 week follow up appointment. Additionally a subcohort of 30 consenting participants were supplied with a kit containing sufficient DBS cards, finger lancets and pre-paid postal envelopes to provide weekly DBS from home during the 6 week follow up. Weekly DBS samples were dispatched to the NICSM laboratory by postal service in business reply envelopes, which contained a ziploc sealable biohazard pouch with silica gel desiccant. All DBS cards were logged on receipt and stored at − 80 °C until analysis. EDTA blood tubes were centrifuged on the day of collection at 5000 g, 500 ul plasma aliquots placed in autoclaved Eppendorf tubes and stored at – 80 °C until analysis.

### Determination of CRP levels

Reference CRP concentration was quantified for whole blood samples (processed to plasma by centrifugation shortly after collection) using a high-sensitivity particle enhanced immunoturbidimetric assay run on a Cobas 8000 c701 system (LOD 0.15 mg/L; measuring range 0.3–20 mg/L; Roche Diagnostics, UK; dilutions and retests made on samples exceeding the assay range) at the Department of Biochemistry, Altnagelvin Hospital, UK (data subsequently referred to as ‘reference hospital’ in text or *Ref Hosp* in Figures). DBS were processed on the day of analysis by punching standardised 3 mm paper discs from the centre of each DBS sample into an Eppendorf tube, then rehydrated in 40 ul of 20 mM Tris–HCl (pH 7.5), 50 mM NaCl, with gentle agitation for 5 min at ambient room temperature (23 °C). The CRP concentration of DBS and plasma samples were determined in duplicate by a Quantikine enzyme‐linked immunosorbent assay (ELISA; LOD 0.022 ng/ml; measuring range 0.8–50 ng/ml) (R&D Systems Inc. Minneapolis, USA) along with quantitative controls (QC70) and standards. All assays and sample dilutions were performed according to manufacturers’ recommendations (data subsequently referred to as ‘DBS ELISA’ or *DBS EL* and ‘plasma ELISA’ or *Plasma E*L, respectively). ELISA plates were read at 450 nm with 540 nm wavelength correction on an Epoch microplate reader in NICSM (BioTek Instruments Inc., USA). The ELISA readings were converted to mg/L and for DBS eluent were corrected based on previous studies indicating that a standardised 3 mm filter paper disc could be saturated with 3.0 ul of blood^16^.

### Statistical analysis

Parametric, two tailed student t-tests were performed comparing demographics (age and other numerical measures) between ‘flare’ and ‘no flare’ patient groups (Table 1). Gender distributions of the two groups were compared using two-sample proportion test. *P* value of < 0.05 was considered a statistically significant difference in all analyses.

**Table 1:** RADAR study cohort demographic information.

	Flare (1) n = 67		No flare (2) n = 25		Total n = 100		t-test * P* value (1) vs. (2)
Female, n (%)	49	(73%)	13	(52%)	68	(68%)	–
Age, mean (SD), years	56.3	(11.7)	59.0	(13)	57.2	(12.6)	ns
Disease Duration, mean (SD), years	6.0	(3.8)	6.5	(4.5)	6.0	(3.9)	ns
Erythrocyte Sedimentation Rate, mean (SD), mm per hr (T0)	16.1	(15.8)	12.1	(15.2)	15.5	(15.5)	ns
Patient Assessed Pain (T0)	55.4	(28.)	32.2	(28.1)	47.3	(30.2)	< 0.01
Patient global assessment of disease activity (PGA) (T0)	58.2	(26.1)	39.3	(28.3)	50.8	(30.2)	< 0.01
C-reactive Protein (T0), mean (SD), mg/L	8.0	(10.7)	7.7	(11.9)	7.9	(0.8)	ns
Change in CRP (T6–T0)	13.1	(88.8)	1.0	(10.8)	9.3	(73.7)	ns
Disease Activity Score in 28 joints (DAS28-ESR), mean (SD) (T0)	3.9	(1.5)	3.1	(1.4)	3.6	(1.5)	0.025
DAS28-ESR (T6–T0)	0.4	(1.2)	0.2	(1.1)	0.4	(1.2)	ns

Patients are grouped depending on disease flare reported during the 6-week follow up period; 8 of the 100 participants did not provide their flare status. Baseline mean values are indicated by (T0), whereas changes over the period of the study are indicated by (T6–T0) values. Patient assessed pain and disease activity were recorded by participants at T0 and T6 on a 0–100 visual analogue scale. Two sample parametric, two tailed t tests were performed for baseline DAS28, baseline CRP, patient assessed pain and disease activity. Two sample non parametric, one tailed t tests were performed for change in DAS28 and change in CRP data.

*SD* standard deviation, *ns* no significant difference.

Bland–Altman analysis (Fig. 2) and plots were used to compare paired sample CRP results, and findings are expressed as mean bias differences with 95% limits of agreement, using GraphPad Prism version 8.2.1. The plots with the 95% limits of agreement show the means of CRP values between each sample method plotted on the horizontal axis and differences between methods plotted on the vertical axis.

Weighted Deming regression was performed to estimate the systematic bias and its confidence interval between the reference whole blood and DBS or plasma CRP values, using NCSS Statistical Analysis and Graphics software version 19.0.3. Outliers, defined as points above or below the 95% limits of agreement on each Bland–Altman scatterplot were removed prior to Deming regression. Regression coefficients and predicted values were calculated using the formulas given in Linnet^17^. The standard errors of the regression coefficients and predicted values are calculated using the combined jackknife leave-one-out method (n − 2 degrees of freedom is used for the jackknife standard error estimates; Fig. 3E,F)^18^. The nonparametric Spearman correlation coefficients (r) were calculated with a two tailed test of significance.

In order to assess the sensitivity and specificity of DBS CRP to accurately detect disease flare, area under the curve (AUC) and change from baseline CRP metrics were calculated from the longitudinal data of the weekly DBS participant subcohort. CRP metrics were calculated by different methods (see Fig. 4): (i) by using all weekly data points to construct an accurate continuous data AUC value, or (ii) by using baseline and 6 week data points to estimate a minimal data AUC value or (iv) only assigned positive flare status if DBS CRP concentrations exceeded 10 mg/L. A threshold AUC value of 35 mg week/L (the mean value in patients reporting no flare) was used assign positive ‘flare’ status to (i) and (ii). A Mann–Whitney test was used to compare significance of any difference between metrics for flare and no flare subcohorts.

### Ethics approval and consent to participate

Office for Research Ethics Committees Northern Ireland (ORECNI) (16/NI/0039), Ulster University Research Ethics Committee (UREC) (REC/16/0019) and WHSCT (WT/14/27) approvals were obtained for the study.

### Consent for publication

Informed consent was obtained for all participants in the study, allowing for publication of anonymised clinical data.

## Results

### Patient demographics

Table 1 shows the demographic information of participants in the study grouped by the incidence of self-reported flares. The mean age of the study participants was 57.2 ± 12.6 years, with a mean disease duration of 6.0 ± 3.9 years. 67% of all participants reported flares during the 6 week study, with significantly higher mean baseline pain (on a visual analogue scale of 0–100^19^) reported in the ‘flare’ group, 55.4 ± 28.0, versus the ‘no flare’ group, 32.2 ± 28.1 (*p* = 0.0007). There was no significant difference in gender distributions between the two groups (*p* = 0.094). Both mean patient-global assessment (PGA)^20,21^ (‘flare’ 58.2 ± 26.1 vs. ‘no flare’ 39.3 ± 28.3; *p* = 0.0034) and mean DAS28-ESR^22,23^ (‘flare’ 3.9 ± 1.5 vs. ‘no flare’ 3.1 ± 1.4; *p* = 0.0251) were significantly higher in ‘flare’ subgroups at baseline. There was no statistically significant association between flare incidence and the duration of disease, baseline CRP or ESR (*p* > 0.05). Absolute changes in CRP or DAS28 over 6 weeks were not significantly different in participants who reported disease flare.

### CRP assay data comparison

**Figure 1:**
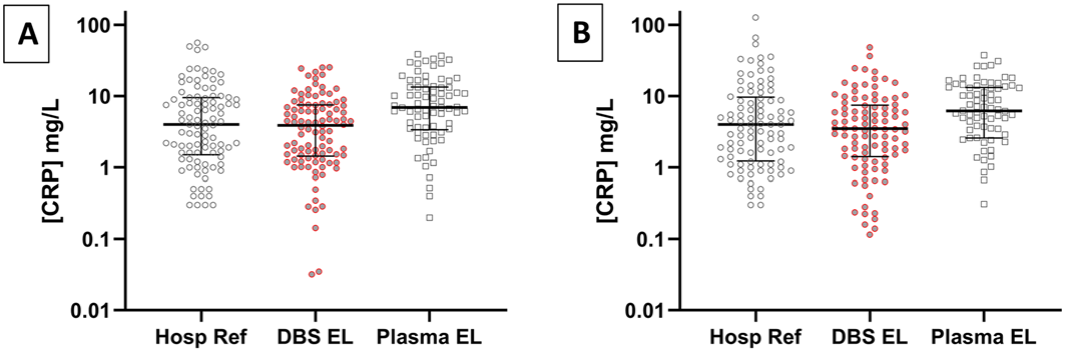
Distribution of CRP samples values recorded. The distribution of CRP measurements by immunoturbidimetry analysis of whole blood (Hosp. Ref.) and ELISA testing of dried blood spot (DBS EL) and plasma (Plasma EL), in (**A**) baseline and (**B**) 6 week samples for n = 100 RADAR study participants. Log10 scale of C-reactive protein concentration in mg per ml. Error bars represent the 25th and 75th percentile with median indicated at centre line.

Baseline reference hospital immunoturbidimetry CRP concentrations of plasma samples have a similar median, 4.00 mg/L to paired DBS ELISA samples, 3.88 mg/L, though lower than the plasma ELISA median, 6.95 mg/L (Fig. 1A). Baseline measurement ranges for each assay were 55.80 mg/L, 25.42 mg/L and 38.61 mg/L, respectively. 6 week CRP median data was similar for reference hospital assay, 4.00 mg/L, DBS ELISA, 3.49 mg/L and plasma ELISA, 6.20 mg/L. 6 week measurement ranges for each assay were 126.20 mg/L, 48.25 mg/L, 37.63 mg/L, respectively (Fig. 1B).

### Sample method comparison

**Figure 2:**
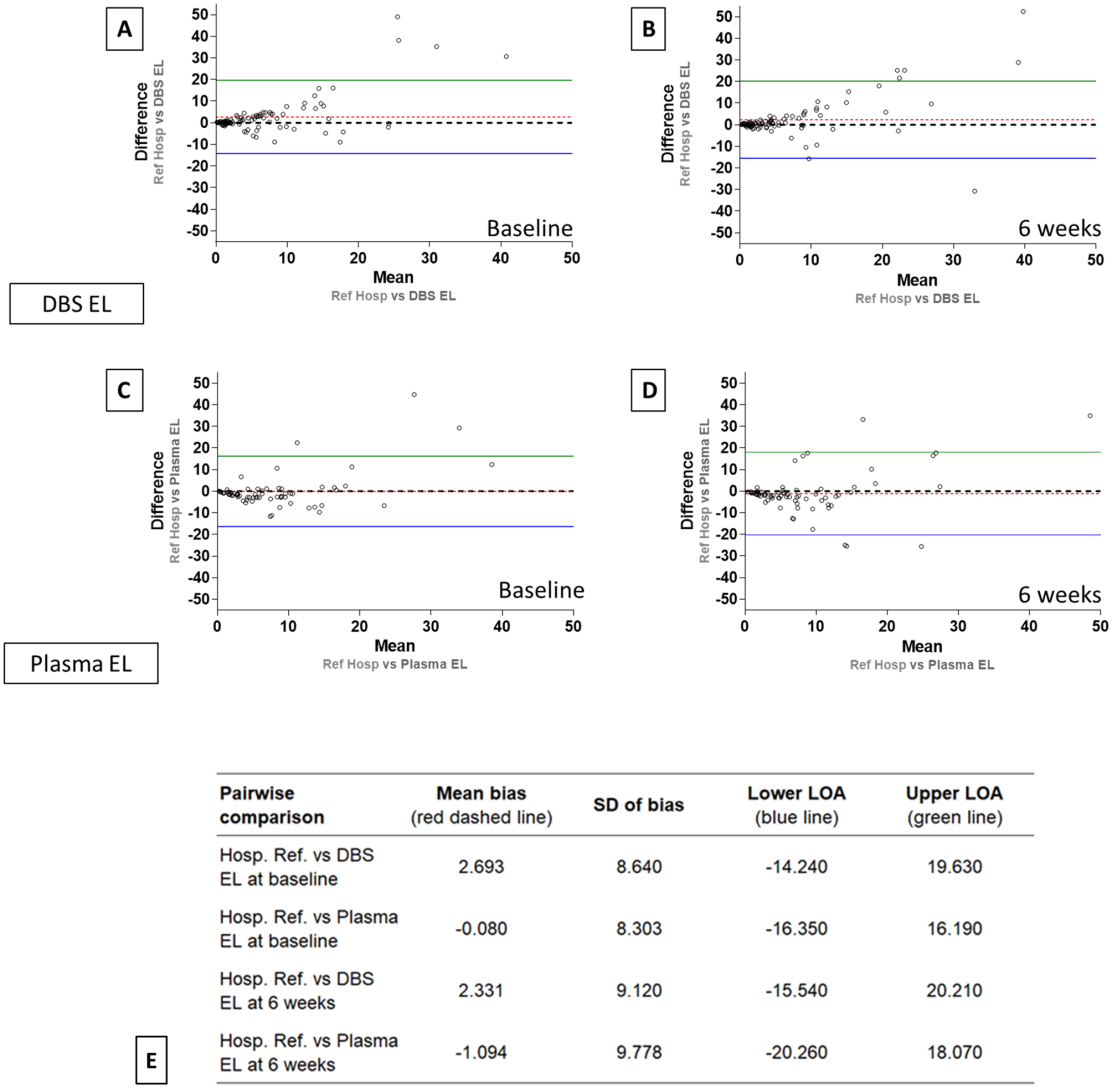
Bland–Altman method comparison plots. Bland–Altman analysis comparing the measurement of CRP using the two different sampling methods across n = 100 RADAR study participants. (**A**) and (**B**) compare agreement between immunoturbidimetry analysis of whole blood (Hosp. Ref.) and ELISA testing of dried blood spot (DBS EL), at baseline and 6 weeks as labelled. (**C**) and (**D**) compare agreement between immunoturbidimetry analysis of whole blood (Hosp. Ref.) and plasma (Plasma EL), at baseline and 6 weeks as labelled. The black dashed line represents the mean, the red dashed line represents the average bias (or the average of the differences), while the upper green and lower blue lines represent ± 1.96 standard deviation. (**E**) The statistical parameters of the Bland–Altman plots, comparing levels of agreement and bias between sample methods relative to the reference hospital immunoturbidometry method are shown. The level of agreement (LOA) line is calculated as mean difference ± 1.96 multiplied by standard deviation. Points contained within the LOA lines denote good agreement between the two methods.

**Figure 3:**
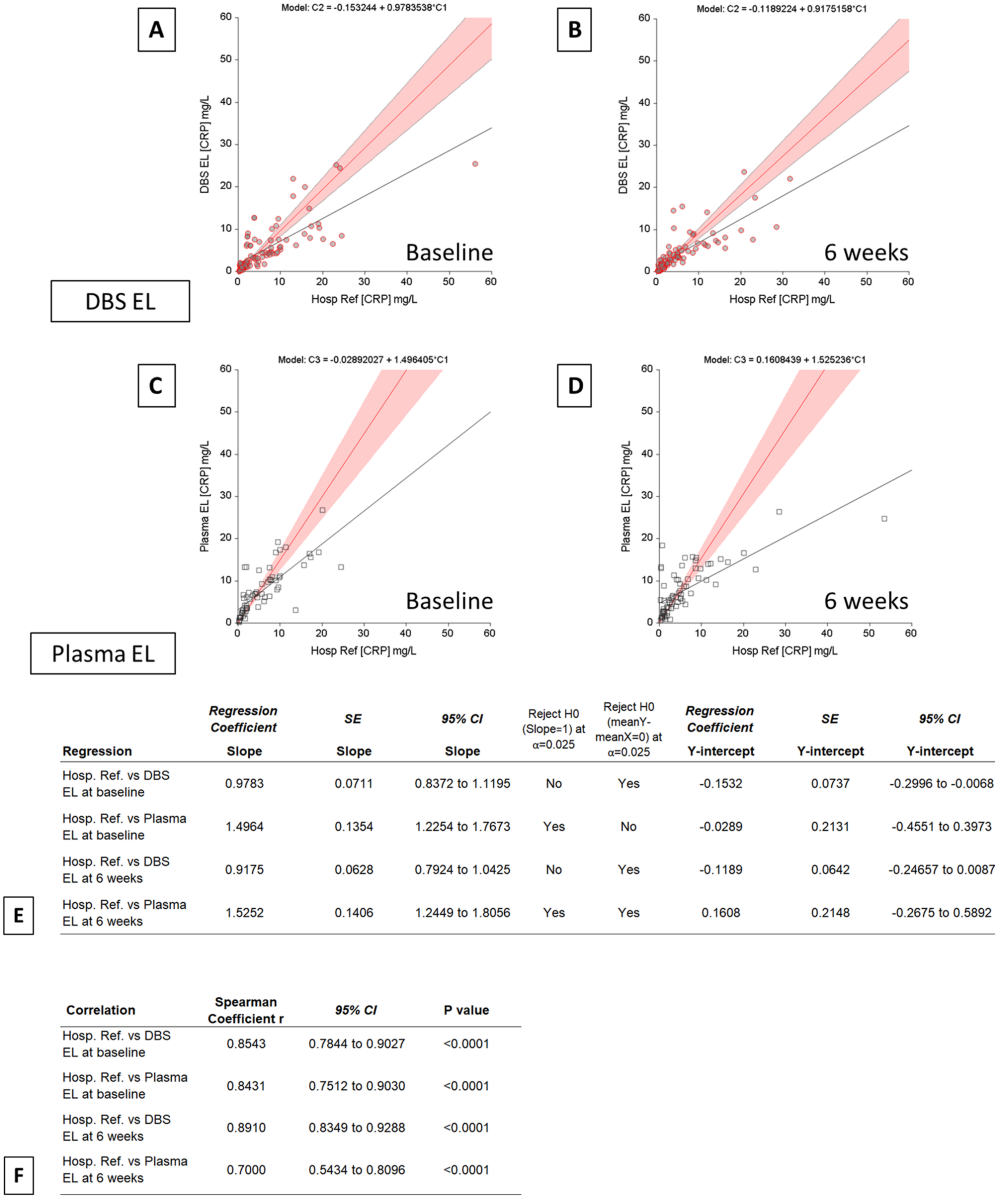
Deming regression method comparison analysis. Weighted Deming regression analysis comparing the measurement of CRP using the two different sampling methods across n = 100 RADAR study participants. Graphs compare systematic differences between immunoturbidimetry analysis of whole blood (Hosp. Ref.) and ELISA testing of dried blood spot (**A**,**B**; DBS EL) and plasma (**C**,**D**; Plasma EL), at baseline and 6 weeks. The red line represents the Deming regression line, the black line represents a simple linear regression line and the red shaded area the 95% confidence intervals. (**E**) The statistical parameters summarised from the Deming regression analysis compare systematic differences between immunoturbidimetry analysis of whole blood (Hosp. Ref.) and ELISA testing of dried blood spot (DBS EL) and plasma (Plasma EL), at baseline and 6 weeks. (**F**) Spearman correlation coefficients for each sample data comparison. *Cl* confidence limits of mean difference; *SE* standard error.

Figure 2 shows the Bland–Altman plots, comparing CRP measurements from DBS ELISA and plasma ELISA to reference hospital assay data. Baseline DBS ELISA yielded slightly higher values with a mean positive bias of 2.693 ± 8.640 (95% limits of agreement − 14.24 to 19.63%; Fig. 2E). The 6 week DBS ELISA samples showed a similar positive bias of 2.331 ± 9.120 (95% limits of agreement − 15.54 to 20.21%). The plasma ELISA data indicate modest negative biases of − 0.080 ± 8.303 and − 1.094 ± 9.778 for baseline and 6 week samples, respectively. The Bland–Altman difference vs. average plots cone shaped distributions observed for both DBS and plasma indicate that the measurement error between the methods are proportional but not constant and that a weighted Deming regression approach was required. Deming regression revealed good agreement between the DBS ELISA method and the reference hospital assay (Fig. 3A,B) with baseline slope of 0.978 and intercept -0.153 and 6 week slope of 0.918 and intercept − 0.119 (Fig. 3E). Plasma ELISA did not agree with the reference hospital data with slopes of 1.496 and 1.525 observed for baseline and 6 week samples, respectively.

### Flare detection during follow up

**Figure 4:**
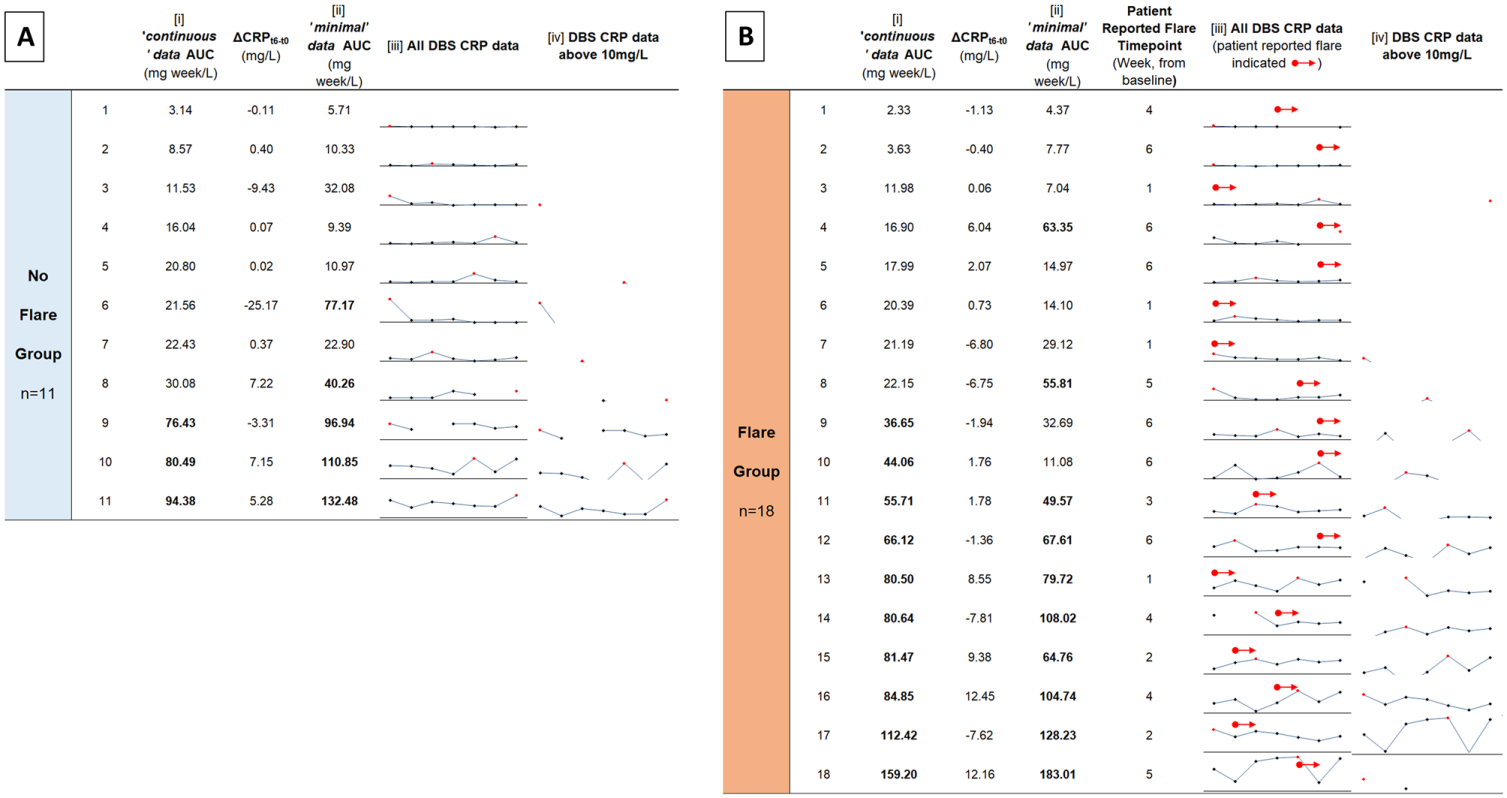
Longitudinal DBS CRP measures in home based arthritis patients. Tables summarising area under the curve (AUC) measures and change (ΔCRP_t6–t0_; 6 week [CRP]—baseline [CRP]) in DBS CRP concentration over the 6 week monitoring period for a subcohort of: (**A**) 11 participants who did not report a ‘flare’ and (**B**) 18 participants who did report a flare (1 individual did not provide their flare status). AUC was calculated by two different methods: in column (i) by using all weekly data points to construct an accurate *continuous data* AUC value, or in column (ii) by using baseline and 6 week data points to estimate a *minimal data* AUC value. Sparklines indicate the DBS CRP concentration of each participant over the 6 week period, such that (iii) CRP concentrations from weekly DBS are indicated by individual data points plotted on the same scale (with red data point indicating high point), or (iv) with only DBS CRP concentrations above 10 mg/L indicated. The week in which patients reported flare is listed and indicated by a red arrow in column (iii) sparklines. A threshold of 35 mg week/L was used assign positive ‘flare’ status, indicated in bold in columns (i) and (ii). Only participants with sparkline data visible in column (iv) were assigned positive flare status.

Area under the curve (AUC) values were used to assess whether the ‘*continuous*’ weekly DBS CRP readings influenced the sensitivity or specificity to correctly identify flare, compared to a typical clinical instance where ‘*minimal*’ baseline and 6 week data only were available (Table 2 and Fig. 4).

**Table 2:** Disease flare detection from weekly DBS CRP.

Longitudinal DBS CRP metric	No Flare Mean (n = 11)	Flare Mean (n = 18)	Mann–Whitney * P* value	TP	TN	FP	FN	Sensitivity (%)	Specificity (%)	PPV (%)	NPV (%)
[i] 'continuous' AUC (mg week/L)	35.04	44.65	0.54	9	8	3	8	52.9	72.7	75.0	50.0
[ii] *“minimal'* AUC (mg week/L)	49.92	49.58	0.73	9	6	5	8	52.9	54.5	64.3	42.9
[iv] CRP data above 10 mg/L	–	–	–	11	3	8	6	64.7	27.3	57.9	33.3

Data summarising the sensitivity and specificity of each metric of longitudinal DBS CRP concentration, (i), (ii) and (iv), to detect flare is shown for a subcohort of 30 participants who sent week DBS samples from home over the 6 week monitoring period. 11 participants did not report a ‘flare’ and 18 participants did report a flare (1 individual did not provide their flare status). AUC was calculated by three different methods (see Fig. 4): (i) by using all weekly data points to construct an accurate *continuous data* AUC value, or (ii) by using baseline and 6 week data points to estimate a *minimal data* AUC value or (iv) only assigned positive flare status if DBS CRP concentrations exceeded 10 mg/L. A threshold of 35 mg week/L was used assign positive ‘flare’ status to (i) and (ii).

*TP* true positive, *TN* true negative, *FP* false positive, *FN* false negative, *PPV* positive predictive value, *NPV* negative predictive value.

Neither method reached statistical significance. The specificity of continuous AUC data (72.7%) to identify flares, was greater than minimal AUC data (54.5%). Sensitivity was the same for both AUC metrics (52.9%). Sensitivity was increased (64.7%), although specificity was reduced (27.3%), when data above a clinically relevant threshold of 10 mg/L was considered. The positive predictive value or precision of the *continuous* AUC data (75%) was also improved, versus the *minimal* AUC data (64.3%). Negative predictive value was also greater for *continuous* data (50%) versus the *minimal* AUC data (42.9%).

## Discussion

The central aim of this study was to establish if CRP values measured from DBS samples using an ELISA agree with those from a conventional plasma sample assayed by a hospital reference immunoturbidometric method. This is the first study exploring this approach in a rheumatoid arthritis population with active disease.

### Method comparison

The Bland–Altman and Deming regression plots in this study show that there is reasonable agreement between the DBS and reference methodologies, especially in the low to mid-range CRP values. The calibration of the standard curve in ELISA does not cope as well with very high levels of CRP as the immunoturbidometric method does^24^. The majority of DBS ELISA measurements (91% in the baseline samples and 85% in the 6 week) lie between the limits of agreement lines (Fig. 2), indicating reasonable agreement between the two collection and assay methods. The weighted Deming regression coefficients for the values within the limits of agreement, which correct for systematic measurement error, indicate good agreement in particular between the DBS ELISA and reference methods.

The imperfect spearman correlation coefficients indicate a proportional error that might have originated from incomplete recovery of CRP in the ELISA methods, particularly with frozen plasma. The non-zero intercepts observed likely reflect a systematic error such as insufficient volume correction in DBS.

The normal probability plots (Supplementary Fig. 1) indicate how closely the two data sets agree, when the two cumulative distributions are plotted against each other, whilst also highlighting any skewness of the distribution. If the two measurements showed perfect agreement with each other, then the plotted data would fall on an ideal-diagonal line. The largest and most consistent skew was observed from the 75th percentile and above, therefore indicating that the higher CRP values measured by ELISA did not show good agreement with the immunoturbidimetry data.

### Flare detection

Our data does not suggest that rises in CRP alone can predict an impending flare in RA patients, but that longitudinal CRP measures can help to confirm biochemical evidence of a flare which can be used by clinicians alongside clinical measures such as tender and swollen joint counts. Continuous weekly CRP data, collected while study participants were at home, was able to identify flare with greater specificity, than minimal data taken at baseline and follow up hospital appointments. The occurrence of flares often prompts an escalation in therapy^25^. In the current study, 16.4% of patients who experienced flare had a change in treatment (addition or removal) and 6% had treatment dose modified at 6 weeks. Whereas in those without flare, 4.5% had a change in treatment and 9% a dose modification. With further sample and analytical refinement, a DBS approach could offer new opportunities to optimally suppress chronic inflammatory episodes, and reduce long term morbidity and mortality risk to people with RA.

### Clinical implications

The decision to treat inflammation in RA can be initiated when mean changes in CRP of 3 mg/L or more distinguish active flare^26^. Thus, changes in higher plasma CRP concentrations beyond the capability of the ELISA method should not adversely influence the course of treatment.

If a clinician were to attempt to identify active flares using a DBS CRP assay, the 95% limits of agreement (using 6 week Bland–Altman parameters) between the two methods at the active disease cut-off of 10 mg/L would be 8.45–12.02 mg/L^27,28^. This could be tolerable from a clinical perspective as the variance observed for reference readings below 20–25 mg/L is not appreciable and the positive bias shown with DBS sample readings may compensate to some degree for under measurement. Though the imprecision may mean a small number of patients with borderline flare may be overlooked, it would still allow patients with active disease clearly above a 10 mg/ml CRP threshold to be accurately identified.

### Limitations

The agreement between DBS and reference methods was acceptable with CRP concentrations in the low to mid-range, but DBS CRP readings for samples with reference values above 25 mg/L were imprecise, with increased variance beyond the 95% confidence limits. It is possible that the use of ELISA assays may have caused a systematic bias, as both ELISA assays showed imprecision with higher levels of CRP. Another potential source of error with DBS samples is changes in haematocrit due to inaccurate volume correction estimates, though dried plasma solutions may mitigate against this^29–31^. It is also likely that using the patient’s subjective ‘self-report’ of flare will not always distinguish an inflammatory flare from an increase of pain due to other causes. However, a recent study comparing patient and clinician reported flares indicates close agreement across 79–93% of joints, particularly on swollen joints^32^.

### Conclusions

The good agreement indicated between DBS and the reference method at low to mid-range CRP could pave the way for development of self-sampling blood collection devices as part of novel remote monitoring services available to individuals living with chronic diseases such as rheumatoid arthritis.

## Supplementary information

Supplementary Legend.

Supplementary Figure 1.

## Data Availability

The datasets used and/or analysed during the current study are available from the corresponding author on reasonable request.

## Abbreviations

RA: Rheumatoid arthritis
CRP: C-reactive protein
DBS: Dried blood spot
ELISA: Enzyme linked immunosorbent assay
ESR: Erythrocyte sedimentation rate
DAS28: Disease activity score across 28 joints
WHSCT: Western Health and Social Care Trust
RADAR: Remote Arthritis Disease Activity MonitoR
DMARD: Disease modifying anti-rheumatic drug
ORECNI: Office for Research Ethics Committees Northern Ireland
UREC: Ulster University Research Ethics Committee
NICSM: Northern Ireland Centre for Stratified Medicine
AUC: Area under the curve
T0: Baseline time point
T6: 6 Week time point
