# Supplementary material for: Assessment of a dried blood spot C-reactive protein method to identify disease flares in rheumatoid arthritis patients
Source: Sci Rep. 2020 Dec 3;10:21089. doi: 10.1038/s41598-020-77826-0 (PMC7713120; doi:10.1038/s41598-020-77826-0)
Supplement: Supplementary file 1 — Supplementary Legend. [file 41598_2020_77826_MOESM1_ESM.docx]

**Supplementary Figure 1 Normal probability plots of optimized residuals.**

The probability plots of residuals from weighted Deming regression indicate the skewness of the distribution when the difference in CRP measurements between the reference hospital and the indicated method: **A** and **B** dried blood spot (DBS EL), **C** and **D** plasma (Plasma EL), at baseline and 6 weeks respectively.
