# Supplementary figures and images for: Assessment of a dried blood spot C-reactive protein method to identify disease flares in rheumatoid arthritis patients
Source: Sci Rep. 2020 Dec 3;10:21089. doi: 10.1038/s41598-020-77826-0 (PMC7713120; doi:10.1038/s41598-020-77826-0)

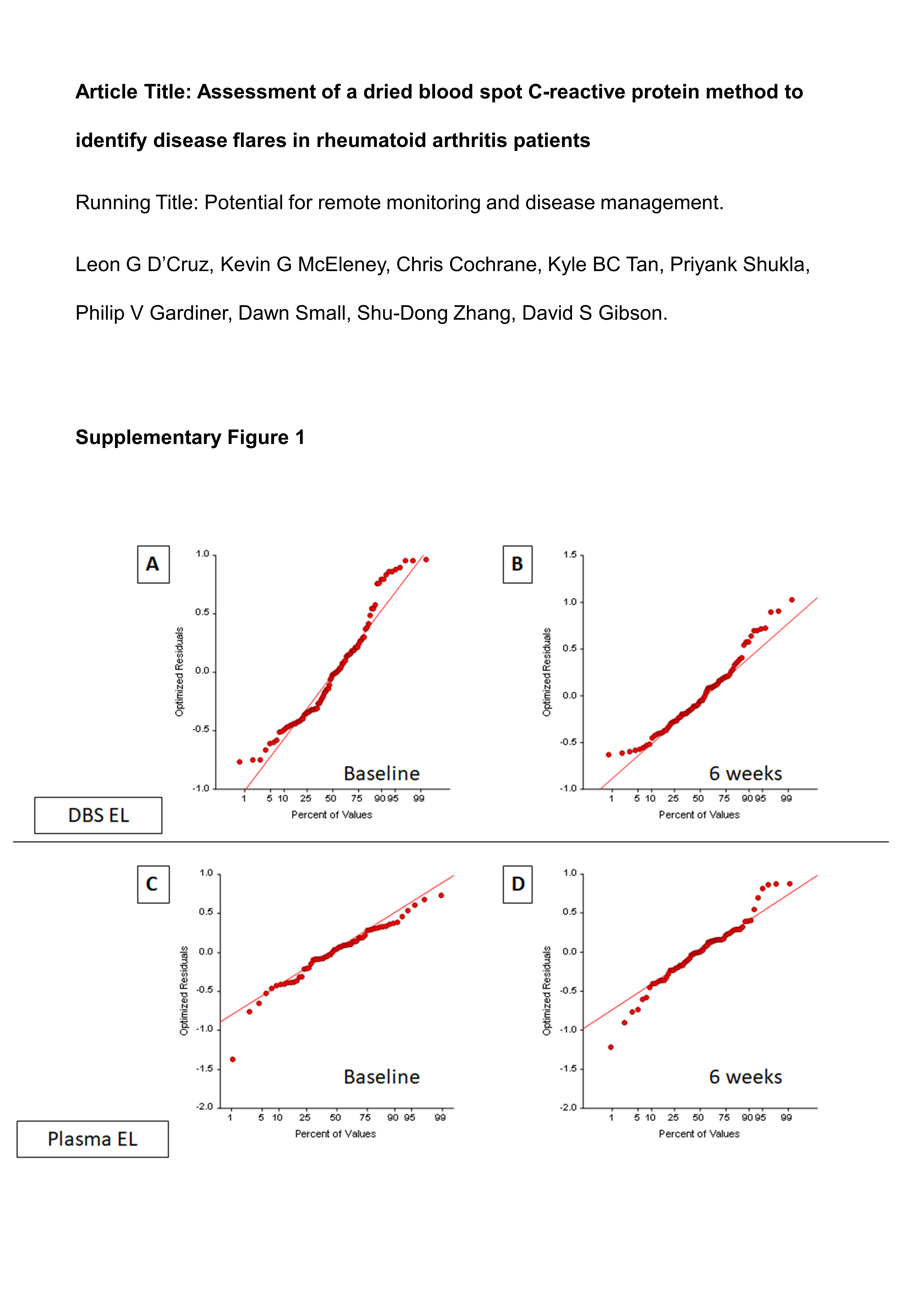

Supplement: Supplementary file 2 — Supplementary Figure 1. [file 41598_2020_77826_MOESM2_ESM.tif]
